# Psychometric properties of the Tilburg Pregnancy Distress Scale-Persian version (TPDS-P)

**DOI:** 10.1186/s12884-021-04078-9

**Published:** 2021-09-06

**Authors:** Solmaz Pishahang, Sevil Hakimi, Solmaz Vatankhah, Saeideh Ghaffarifar, Fatemeh Ranjbar

**Affiliations:** 1 Research Center of Psychiatry and Behavioral Sciences, Tabriz University of Medical Sciences, Tabriz, Iran; 2 School of Nursing and Midwifery, Tabriz University of Medical Sciences, Tabriz, Iran; 3 Medical Education Research Center, Health Management and Safety Promotion Research Institute, Tabriz University of Medical Sciences, Tabriz, Iran

**Keywords:** Tilburg Pregnancy Distress Scale, Pregnant Women, Psychometric Properties

## Abstract

**Background:**

Given the importance of screening pregnant women’s distress, it was intended to investigate the psychometric properties of the Persian version of the Tilburg Pregnancy Distress Scale (TPDS-P) for screening pregnancy distress.

**Methods:**

This methodological psychometric study was conducted with participation of 360 pregnant women. The TPDS was translated into Persian. Factor analysis was used to investigate the construct validity. The results of the correlation test between the results of the two questionnaires, Depression Anxiety Stress Scales-21 (DASS-21) and TPDS-P, were used to determine the criterion validity of TPDS-P. Internal consistency of the items was calculated by the Cronbach's alpha coefficient. Stability of the results was examined by test-retest method and Intra-class Correlation Coefficient (ICC) was calculated. Examining the structure of the factors derived from exploratory factor analysis, fitness of the model was done through confirmatory factor analysis. Statistical analysis was done using SPSS software.

**Results:**

Kaiser-Meyer-Olkin (KMO) was 0.846 (p = 0.001). Sixteen items of TPDS-P accounted for 51.42 percent variances. The TPDS-P exhibited appropriate fitness. There was poor to moderate but significant direct correlation between the subscales of DASS-21 and TPDS-P. Cronbach’s alpha coefficient of the TPDS-P was 0.81 and ICC was 0.70.

**Conclusions:**

TPDS-P, with appropriate validity and reliability, can be used as a practical scale to evaluate women's distress during pregnancy in Farsi-speaking societies.

## Background

Pregnancy and delivery are physiological events in a woman's life that, due to changes in physical conditions, family and workplace roles, and sometimes feelings and attitudes toward motherhood; require bio-psychosocial adjustment by the pregnant woman and her family [1]. This can be due to anxiety and fear of labor pain, lack of trust in the delivery room staff and stress caused by entering the unknown space of the delivery room, which is itself a factor that increases catecholamine, cortisol and epinephrine [2]. These stresses affect the adrenal axes, pituitary-hypothalamus and sympathetic nervous system, leading to endocrine neuronal changes and as a result to cortisol and adrenocorticotropin secretion. Cortisol and adrenocorticotropin lead to an increase in plasma cortisol levels, which in turn increases maternal stress during pregnancy [3]. Pregnancy related distress means the pregnant women's worry and concern about many different issues such as the health of the fetus, relationship with the spouse and others, changes in the body through delivery and pregnancy and the newborn's health [1]. Recent investigations have shown that more than 80% of pregnant women experience some degrees of psychosocial stress, and 20% of women experience severe stress [4]. In addition, increased cortisol levels rapidly results in problematic fetal growth , developmental disorders and reduces fetal heart rate [3] . Decreased Apgar score, lactation problems, low birth weight, late attachment between mother and baby, and increased cesarean section rate are other problems associated with increased stress and anxiety levels in the mother [1, 2, 5].

Anxiety and depression are two concepts which are closely related. Traditional tools for distinguishing between anxiety and depression have failed. Hamilton’s tools for anxiety and depression have been shown to overlap in content and they are related to each other [6]. The severity and prevalence of anxiety increase with increasing gestational age [7]. The reason can be strongly attributed to the blood level of progesterone which increases by gestational age [8]. Pregnancy anxiety reduces maternal and fetal attachment, leading to postpartum depression [9]. If postpartum depression left untreated, it causes many problems such as attachment disorders, developmental disorders, suicide and others [10]. Decreased milk production and secretion, irritability, unstable conditions in the newborn and decreased mental development of the child at the age of two are other complications of postpartum depression [11]. In general, stress, depression, or anxiety during pregnancy not only have a major impact on women's health and their quality of life, and increase the risk of midwifery complications, but also may affect the child's neurological and emotional development [12] and ultimately predict his or her health problems [13].

Given the consequences of depression, stress and anxiety during pregnancy on both maternal and fetal health, the need for measurement of psychological functioning during pregnancy and screening the vulnerable women in an effective way is deeply felt [12]. To measure depression, anxiety and stress in the general population, various instruments, such as Depression Anxiety Stress Scales-21 (DASS-21), BAI: Beck Anxiety Inventory [14] , Edinburgh Depression Inventory [15] and PSS-14: Perceived Stress Inventory are used by many researchers [16]. These questionnaires may be used to measure gestational distress but are not specific to assess women's distress during pregnancy [12]. Studies have shown that only 20% of gynecologists routinely screen for anxiety disorders in their clinical work [17] and less than half of them state that they have received sufficient training to diagnose depression during their residency [18].

To determine the level of distress during pregnancy, Victor JM Pop and associates developed and presented a valid scale for examining the specific psychological function of pregnancy named Tilburg Pregnancy Distress Scale (TPDS), which included 22 initial items. Later, its psychometric properties were investigated using exploratory factor analysis. It was resulted in a 17-item TPDS, which was further explored by confirmatory factor analysis and concurrent validity assessment and finally a 16-item scale was developed. Subsequent analysis confirmed that this scale has two subscales of negative affect and partner involvement and it can be a valid and useful instrument for the assessment of pregnancy distress [12].

Due to the considerable prevalence of distress during pregnancy and given the point that there was no tool translated in Persian to measure distress during pregnancy in Farsi speaking countries, it was intended to translate the TPDS in Persian and investigate the psychometric properties (validity and reliability) of the Persian version of the Tilburg Pregnancy Distress scale (TPDS-P) for screening pregnancy distress in women in Farsi-speaking countries.

## Methods

This is a methodological psychometric study in terms of nature and descriptive cross-sectional in terms of implementation method.

### Participants

The population of the present research included pregnant women, under the coverage of the routine pregnancy care provided by healthcare centers in Tabriz, Iran, from October 2017 to March 2018. The inclusion criteria included: age above 18 years, gestational age more than 24 weeks, absence of a chronic disease, absence of high-risk pregnancy (including gestational diabetes or preeclampsia), absence of twin pregnancy, absence of assisted reproductive techniques for pregnancy, and voluntary participation. The exclusion criteria included presence of any intellectual or cognitive problems.

The sample size was calculated to be 360. 160 participants were considered for conducting an exploratory factor analysis (EFA) (5-10 women considered for each of the 16 items of the TPDS-P). 200 pregnant women were also required for running the confirmatory factor analysis (CFA) [19].

### Procedure

The multistage random sampling method was used in the research. First, 15 centers were randomly selected from the list of all healthcare centers in Tabriz using the www.random.org website. Later, the medical records of the pregnant women at the time of sampling were examined. Then a number of medical records were selected from each center (using quota random sampling) regarding to the total number of available files. After giving detailed explanations about the reasons, purposes and methods of the research, the pregnant women with considering gestational age (>24 weeks) were invited to the medical centers via phone call to participate in the study. If agreed, they would be requested to attend the treatment & health centers according to the specified schedules. In that step, they were given comprehensive information about the methods and benefits of the research and the confidentiality of their information. If they agreed to participate in the study, they were reevaluated in terms of the inclusion criteria. If they were eligible, written informed consent for participation would be obtained from them.

The authors of the research obtained permission for the translation of the original scale into Persian and its adaptation and application. The process of translation and adaptation has gone through using the forward-backward translation approach: Two bilingual translators, including a psychiatrist and a reproductive healthcare specialist, separately translated the original version of the scale into Persian. The two translated versions were compared for the identification of any disagreements and ambiguities. After the points of disagreement were revealed and resolved, a single version of the TPDS-P was prepared and translated into English by two native translators with no involvement in the previous process of translation. Finally, a committee composed of the translators, including the first and the corresponding authors compared the new English version of the scale to the original one and eliminated meaning related problems.

### Measures

All the participants completed three questionnaires including a demographic questionnaire, TPDS-P and DASS-21(used for investigation of the criterion validity of TPDS-P), which are described below:

1. Demographic questionnaire: This contained questions on age, marital status, occupation, level of education, and economic status of the participants.
2. TPDS: The original TPDS is composed of two major subscales and a total of 16 items. The first, negative affect subscale contains 12 items regarding the woman’s fear, anxiety, and concerns about pregnancy and postpartum period, and the second partner involvement subscale, contains 4 items regarding her partner’s support during pregnancy. For each of the items, the participants selected one of four options based on their feelings and perceptions of their pregnancies, depending on the severity of their experiences. Scores from 0 to 3 were assigned to the answers, ultimately (0 for *very often*, 1 for *fairly often*, 2 for *now and then*, and 3 for *rarely or never*). In calculation of the final scores, the scores for items 3, 5, 6, 7, 9, 10, 11, 12, 13, 14, and 16 were considered in reverse. The minimum score was 0, and the maximum was 48. Overall, a higher score indicated greater distress. The maximum score for the negative affect subscale was 36, and that for the partner involvement subscale was 12 [1]. The cutoff of a high score on the TPDS and its subscales (= distressed woman), was set at the 90^th^ percentile which resulted in the following cutoff scores: for the overall scale > 17, for its subscale ‘NA’ > 12, and for its subscale ‘PI’ >7 [12].
3. DASS: This is an instrument that provides valid and reliable assessments of three constructs: depression, anxiety, and stress [20]. Both the internal consistency and concurrent validity of DASS and DASS-21 are in an acceptable to excellent range. Furthermore, the 21-item version has several advantages over the full 42-item one, and can therefore be preferred [6]. The original DASS-21, with a total of 21 items, is composed of three subscales: depression, anxiety, and stress. Each subscale contains 7 items, addressing one's conditions over the previous week. A score from 0 to 3 is assigned to each item (3 for *Applied to me very much*, 2 for *Applied to me to a considerable degree*, 1 for *Applied to me to some degree*, and 0 for *did not apply to me at all*). The score for each subscale can range from 0 to 21. The higher the score, the more severe the depression, anxiety, and stress [21, 22] .Since this is the shortened form of the main 42-item scale, the final score for each subscale needs to be multiplied by 2.

This version has been translated into Persian by Asghari and colleagues*.* in 2008, and has exhibited proper psychometric properties (good to excellent) in the Iranian population [20, 22]

### Statistical analysis

The factor analysis method with SPSS was used for the investigation of construct validity of the TPDS-P, and the results of the correlation test between the DASS-21 and TPDS-P were used for investigation of its criterion validity.

For the investigation of reliability, internal consistency of the items was examined. For the measurement of internal consistency, the Cronbach’s alpha coefficient was calculated. For the measurement of stability of the results, the test-retest method was used, and Intra-class Correlation Coefficient (ICC) was calculated. For that purpose, the questionnaire was redistributed to 30 of the participants after 15 days, and correlation coefficient was examined through the ICC test using SPSS. Values above 0.7 were considered acceptable [23].

The Kaiser- Meyer- Olkin (KMO) test was used for the examination of sample size adequacy, and the Bartlett’s test of sphericity was used for the identification of suitability of the data for factor analysis. Factor rotation was carried out through Varimax rotation. Minimum factor load was considered to be 0.3. Eigenvalues on the Scree plot were interpreted for the specification of the number of factors. The naming of the items was done by considering the original questionnaire and the justification of the naming.

After the exploratory factor analysis was completed, the fitness of the exploratory model was assessed through confirmatory factor analysis via AMOS.23 software. The fit indices were used for the examination of the model fitness. The intended indices and their acceptable values for confirmation of the model are as follows: Chi-square statistic (Bollen, 1989) p < 0.05 , Root mean square error of approximation (RMSEA) < 0.08 , Comparative fit index (CFI) (Goffin, 1993) > 0.9 , Normal fit index (NFI) (Bentler and Bonett, 1980) > 0.9 , Tucker-Lewis index (TLI or NNFI) (Tucker and Lewis, 1998) > 0.9 , Incremental fit index (IFI) (Bollen, 1989) > 0.9 .

### Ethical considerations

Before the research began, official permission was obtained from the author of the original version of TPDS, Victor J. M. Pop. Conducting this study was approved by Tabriz University of Medical Sciences (TUoMS) ethical committee. The ethical code was IR.TBZMED.REC.1396.70. All methods were performed in accordance with the relevant guidelines and regulations at TUoMS. Participation in the study was optional and it was done after obtaining written informed consent. Informed consent was obtained from husbands for illiterate women. A total of 4 illiterate women participated in the study and the questionnaires were completed by their husbands. It was possible for the participants to leave the study whenever they wanted. Before the study was conducted, the purposes of the research were explained to the participants. The information of the participants and their families were kept confidential.

## Results

### Demographic characteristics of the participants

In all, 520 pregnant women were invited to participate. 85 women did not agree to participate. 75 women did not return informed consent and 360 women entered the study.

The baseline characteristics of the 360 woman, who entered the study after obtaining informed consent, are presented in Table 1.

**Table 1:** Demographic characteristics of the participants of the study (n=360)

Variable	Frequency	Frequency percentage
**Age(mean)**	28.26 years	5.95
**Number of pregnancies**		
First pregnancy	139	38.6
Second pregnancy	146	40.6
Third pregnancy or more	75	20.9
**Number of childbirths**		
First childbirth	152	42.2
Second childbirth	156	43.3
Third or more childbirth	52	14.5
**Marital status**		
Married	358	99.4
Single	0	0
Widowed	1	0.3
Divorced	1	0.3
**Occupation**		
Housewife	281	78.1
Government job	27	7.5
Freelance job	33	9.2
Retired	0	0
Self employed	19	5.3
**Education level**		
Illiterate	4	1.1
Primary/Secondary School	88	24.4
High School/Diploma	202	56.1
University degree	66	18.3
**Spouse occupation**		
Government job	91	25.3
Freelance job	262	72.8
Retired	1	0.3
Not employed	6	1.7
**Spouse education level**		
Illiterate	5	1.4
Primary/Secondary School	84	23.3
High School/Diploma	185	51.4
University degree	86	23.9
**Home Status**		
Homeowner	134	37.2
Tenant	166	46.1
Living with parents or relatives	60	16.7
**Monthly income status to meet your needs**		
Completely in financial well-being	19	5.3
Living with economizing the budget	120	33.3
Sometimes had financial difficulties	140	38.9
Always had financial difficulties	70	19.4
Complete poverty	10	2.8

### Data analysis

#### Exploratory factor analysis

In this study, the KMO value was 0.846 and the Bartlett’s test of sphericity was conducted to specify whether the obtained correlation matrix was significantly different from zero, which justified application of factor analysis, and it was observed that p < 0.001 (Chi-square = 2468 and Degree of freedom = 120).

**Fig. 1:**
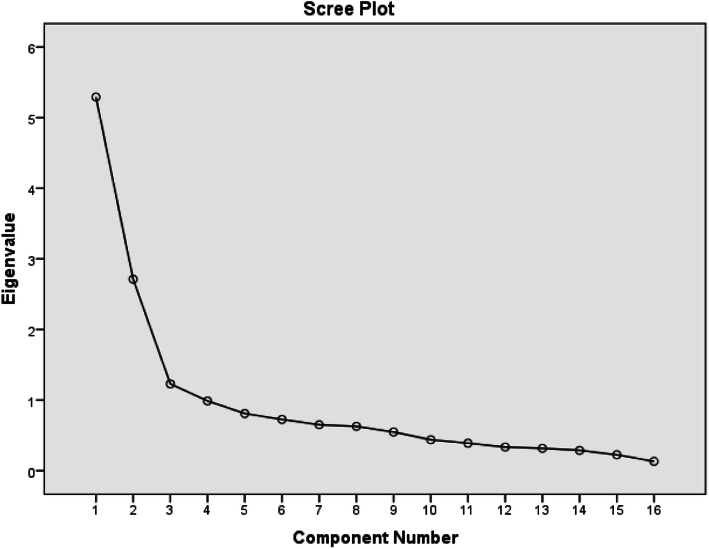
Factor load scree plot of the items for determining the number of extracted factors of the TPDS-P

The Communalities of all 16 items were more than 0.5 in all cases.16 items with eigenvalues greater than 1 were extracted. They accounted for a total of 51.42 percent of all the variances for the two factors. Considering the explanation of 50 percent of the variances by the factors, and using logical interpretability and the result of the scree plot (Fig. 1), and most importantly by considering the original questionnaire, 2 factors were found to be appropriate. Then agreements were made on the naming of each factor based on the original questionnaire.

The two factors were described based on the original questionnaire. The first factor, negative affect, with 11 items accounted for a variance of 30.31%. The second factor, partner involvement, with 5 items explained a variance of 21.1%.

The factor loading values of the items in each subscale are shown in Table 2.

**Table 2:** Factor loading values of the items in each subscale of the TPDS-P

No.	Expressions	Factor loading	
		1	2
**1**	I am enjoying my pregnancy	.024	**.781**
**2**	My partner and I are enjoying the pregnancy together	.029	**.849**
**3**	I worry about my pregnancy	**.768**	-.014
**4**	The pregnancy has brought my partner and me closer together	-.036	**.823**
**5**	I worry about the delivery	.**823**	-.025
**6**	I worry about the health of my baby	**.781**	.029
**7**	I worry about my job once the baby is born	.**122**	.062
**8**	I feel supported by my partner	.111	**.783**
**9**	I worry about our financial status after childbirth	**.375**	.124
**10**	I am afraid I will lose self-control during delivery	**.758**	-.190
**11**	I often worry about choices concerning the delivery	**.738**	-.216
**12**	The delivery is troubling me	**.672**	.187
**13**	I get very tense hearing stories about deliveries	**.788**	-.103
**14**	I am concerned that the physical discomforts of pregnancy might persist after childbirth	**.714**	.068
**15**	I can really share my feelings with my partner	.024	**.762**
**16**	I worry about gaining too much weight	**.321**	.147

### Confirmatory factor analysis

**Fig. 2:**
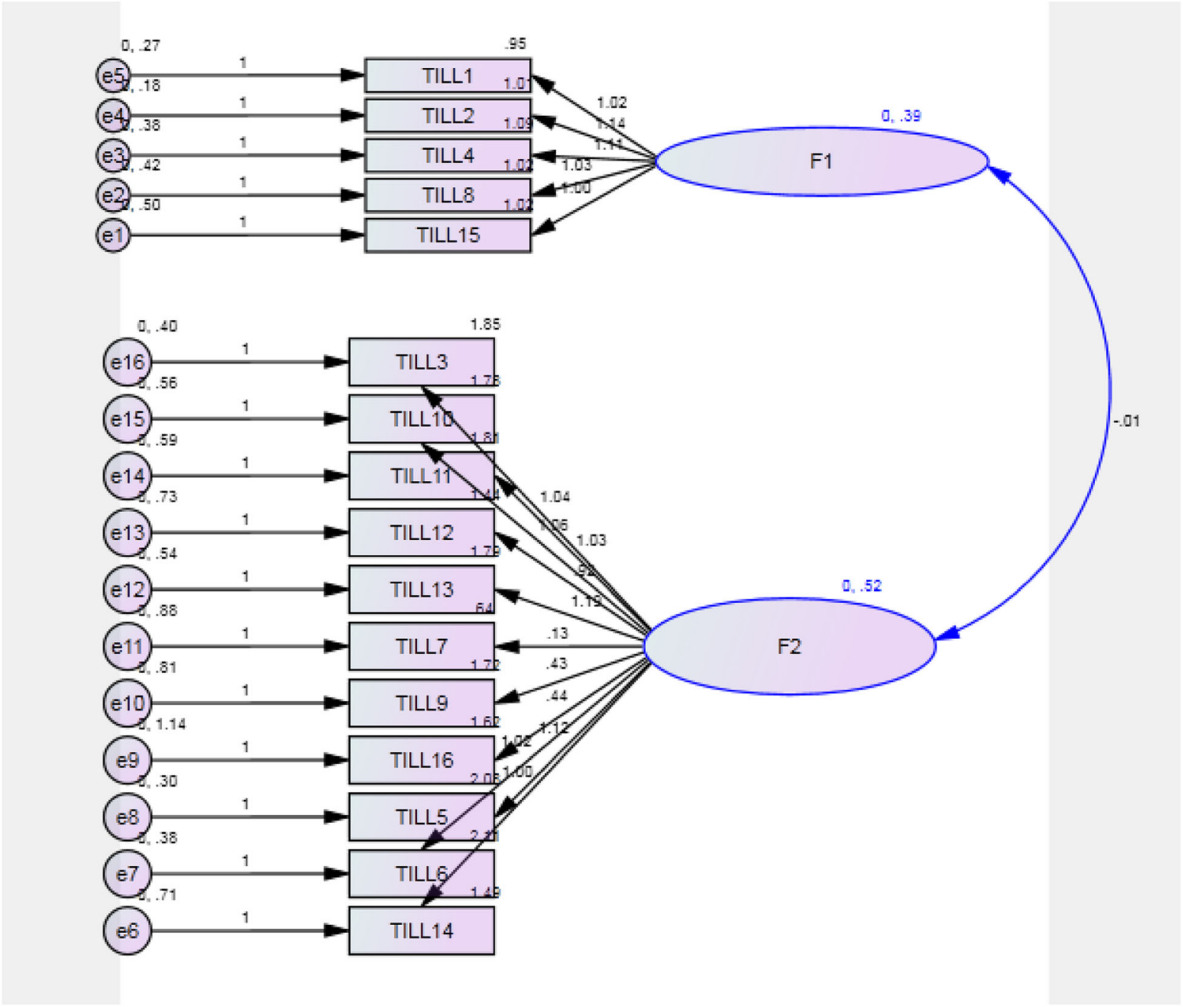
Confirmatory factor analysis graph of the TPDS-P. F1: partner involvement. F2: negative affect

The fit indices of the theoretical model (NNFI > 0.9, CFI > 0.9, NFI > 0.9, IFI > 0.9, and RMSEA < 0.08) were acceptable; hence, the translated questionnaire exhibited appropriate fitness in terms of confirmatory factor analysis. The fit indices of the model are presented in Table 3 and Fig. 2).

**Table 3:** Confirmatory factor analysis results of the TPDS-P

Index	Desirable score	Obtained score
Chi-square statistic	p < 0.05	< 0.001
Comparative fit index (CFI)	> 0.9	0.927
Normal fit index (NFI)	> 0.9	0.937
Tucker-Lewis index (TLI or NNFI)	> 0.9	0.941
Incremental fit index (IFI)	> 0.9	0.989

Table 4 shows the correlation coefficient between the subscales of TPDS-P and DASS-21. There is poor to moderate but significant direct correlation between the subscales of the two questionnaires.

**Table 4:** Criterion validity between TPDS-P and DASS-21

DASS-21	TPDS-P			
	Negative affect r (p)		Partner involvement r (p)	
**Anxiety**	0.562	(0.001)	0.317	(0.001)
**Depression**	0.322	(0.001)	0.296	(0.001)
**Stress**	0.560	(0.001)	0.462	(0.001)

Cronbach’s alpha coefficient calculated for the TPDS-P and the Test-retest results are shown in Table 5.

**Table 5:** Cronbach’s alpha coefficient and Test-retest results for the TPDS-P

Variable	Cronbach’s alpha coefficient	Test-retest coefficient r (p)
Tilburg Pregnancy Distress Scale-Persian Version	0.810	0.701 (0.042)
partner involvement subscale	0.866	0.748 (0.021)
negative affect subscale	0.854	0.622 (0.001)

## Discussion

In this study the TPDS was translated in Persian and the psychometric properties of the TPDS-P were evaluated by participation of 360 pregnant women who were referred to health centers. The results of both exploratory and confirmatory factor analyses showed that the TPDS-P has sufficient validity and reliability.

KMO in the present study was higher than the KMO in the original study. The values obtained for the TPDS-P were in line with those for the original one in terms of sample size and degree of freedom [24].

The obtained variance in the present study (51.42%) is acceptable and lies between 40 and 60 percent which is usual in sociological studies [25].

In the present study, in some items, the results of factor loading and rate of answering were different from the results of the original questionnaire studies. None of the items had exhibited a factor loading lower than 0.3 in the original questionnaire [1], despite item 7 in this research (*i.e.*, “I worry about my job once the baby is born”),which was rarely responded by participants. Not answering this question could be because 78.1 percent of the pregnant women in the current study were housewives, who had no additional jobs, so they had thought there was no need to respond to this item. In a similar study conducted by Ertuğrul et al. in Turkey, similar results were found and the loading factor of item 7 was less than 0.3, because 63% of the participants in Turkey were unemployed [1]. Also in a similar study conducted by Volpato et al. in Brazil, the loading factor of this item was less than 0.3, but in Brazilian version, the reason is stated differently. They believed that certain items have received very similar responses (Such as answers often / very often) [26] . But in the present study, we think that the reason is that 78.1% of women were housewives and women working in the public sector had up to 9 months maternity leave. We did not remove this item from translated version. In this regard, knowing the employment status of participants during the use of TPDS-P in the future studies may be of help.

Item 16 was (*i.e.*, “I worry about gaining too much weight”) another item that got a slightly low loading factor (=0.321) . It seems that the Iranian women are not worried about gaining weight after giving birth. In Iranian society, women do not have a diet to lose weight for at least 6 months after giving birth and pay full attention to their newborns. Due to the customs in this community, it was possible to respond negatively to item 16. This is why the loading factor of this item was reduced. Similar results were obtained in the study by Ertuğrul and et al. in Turkey [1].

To assess concurrent validity of the TPDS-P, DASS-21 questionnaire was used. In the present study, the results of the correlation coefficient test between the subscales of TPDS-P and DASS-21 was not strong and was poor to moderate, but it was significant nonetheless, and it seems that if a psychiatric interview and examination was performed, perhaps better results would be obtained . The highest correlation was between anxiety and stress constructs of DASS questionnaire with negative affect subscale of TPDS-P questionnaire. Due to the similar nature of the structures, there is a stronger correlation between the mentioned structures.

The Cronbach’s alpha coefficient of TPDS-P was calculated to be 0.81, having been 0.78 in the original scale, 0.73 in the Brazilian [26], and 0.70 in the Turkish versions of the scale [1], which demonstrates that this scale is suitable to be used in the Iranian society. Moreover, Cronbach’s alpha was obtained to be higher for the examined subscales than the overall coefficient. It was obtained as 0.86 for the partner involvement subscale and as 0.85 for the negative affect subscale, indicating that the scale performs even better in regard to the items that investigate the partner involvement aspects of pregnancy distress.

As for the confirmatory factor analysis, the indices in the analysis of 4 items in the partner involvement subscale were inappropriate, and item 1 (*i.e.*, “I am enjoying my pregnancy”) exhibited a very low factor loading. Given the results obtained from exploratory factor analysis and the higher factor loading of this item under the second factor (partner involvement), factor analysis was made again to include 5 items under the partner involvement factor; therefore, both the factor loading of item 1 and the overall results of confirmatory factor analysis were thus corrected. Therefore, the partner involvement factor contains 5 items, and the negative affect factor contains 11 items. In the original TPDS, the TPDS-PI and TPDS-NA subscales were “marginally correlated (r = .15)” to each other [12] while they were highly correlated in the present study. As it has been recommended by Victor JM Pop and associates, further future research is needed to interview pregnant women to uncover more details about “the woman’s perception of little partner involvement during pregnancy” [12]. Conducting more research in this regard can make the relationship between these two constructs and their constituent items more complete and clear.

## Conclusions

It can be concluded that Tilburg Pregnancy Distress Scale-Persian version (TPDS-P), as a scale with appropriate validity and reliability, can be utilized in the Iranian society for the assessment of the distress among pregnant women. It can also be used as a screening tool due to the ease of its use. Proper use of this tool can prevent over-referral of pregnant women to a psychiatrist and prevent the imposition of additional financial burden on families and the health system.

### Recommendations and limitations of the study

Since most of the pregnant women under investigation in the present study were housewives with no additional job, it is suggested that as many women with other jobs as housewives be included in future studies, so that the scale can be assessed for them as well. Another limitation of this study was the impossibility of conducting a psychiatric interview to determine concurrent validity.

Since the present study was conducted at urban health centers, it is suggested that similar researches be performed on a rural population.

## Data Availability

All the data and materials will be available from the corresponding author upon any reasonable request.

## Abbreviations

TPDS-P: Tilburg Pregnancy Distress Scale-Persian Version
DASS-21: Depression Anxiety Stress Scales-21
SPSS: Statistical Package for the Social Sciences
ICC: Intra-class Correlation Coefficient
KMO: Kaiser-Meyer-Olkin
RMSEA: Root Mean Square Error of Approximation
CFI: Comparative Fit Index
NFI: Normal Fit Index
TLI: Tucker-Lewis Index
NNFI: Non Normed Fit Index
IFI: Incremental Fit Index
